# The effects of exercise training on heart, brain and behavior, in the isoproterenol-induced cardiac infarct model in middle-aged female rats

**DOI:** 10.1038/s41598-022-14168-z

**Published:** 2022-06-16

**Authors:** Kata Tóth, Tamás Oroszi, Eddy A. van der Zee, Csaba Nyakas, Regien G. Schoemaker

**Affiliations:** 1 Department of Neurobiology GELIFES, University of Groningen, Nijenborgh 7, 9747 AG Groningen, The Netherlands; 2 Research Center for Molecular Exercise Science, University of Physical Education, Budapest, Hungary; 3 Behavioral Physiology Research Laboratory, Health Science Faculty, Semmelweis University, Budapest, Hungary; 4 Department of Cardiology, University Medical Center Groningen, Groningen, The Netherlands

**Keywords:** Neuroscience, Physiology, Systems biology, Cardiology, Diseases, Neurology

## Abstract

Women with cardiovascular disease may be more susceptible to concomitant mental health problems, such as depression and cognitive decline. Exercise training has beneficial effects on the cardiovascular system as well as on mental functions. Aim of the present study was to study the effects of exercise training on heart, brain and behavior in the isoproterenol (ISO) model in middle-aged female rats. Twelve months old female Wistar rats were submitted to ISO injections (70 mg/kg s.c., on two consecutive days) or received saline. One week later, rats were assigned to either exercise training (treadmill running) or control handling for five weeks. During the last 7 days, tests were performed regarding depressive-like behavior and cognitive function. Then, rats were sacrificed and heart and brains were dissected for (immuno)histochemistry. ISO-induced cardiac effects were eminent from cardiac fibrosis and declined cardiac function. Exercise training reversed cardiac damage and partly restored ISO-induced cardiac dysfunction. However, ISO treatment could not be associated with neuroinflammation, nor impaired hippocampal neurogenesis or neuronal function. Accordingly, no cognitive impairment or depressive-like behavior were observed. Actually, hippocampal microglia hyper-ramification was observed after ISO. Exercise left neuroinflammation and behavior merely unaltered, and even reduced neuronal function. Our data indicated that the cardiac damage after ISO in middle-aged female rats, and the subsequent beneficial effects of five weeks exercise training on the heart, were not reflected in changes in the brain nor in altered behavior.

## Introduction

Cardiovascular disease is often associated with mental health problems, such as depression and cognitive decline. Major depression was found in 15–20% of cardiovascular disease patients, while even up to 65% reported symptoms of depression^1,2^. Similarly, cognitive decline or dementia was increased (odds ratio of 1.45) in patients with coronary artery disease^3^. Moreover, women were indicated at higher risk for heart-failure-associated depression^4,5^, but higher prevalence of cognitive decline is reported for both women^6^, and men^5^. This reduced mental health is not an innocent bystander in cardiovascular disease since it is associated with increased morbidity and mortality^7,8^. However, the pathophysiology of the heart-brain interaction^4,5^ is far from fully understood. We hypothesize that the inflammatory response necessary for infarct healing can become derailed and reflected in the brain as neuroinflammation, associated with reduced mental health^9^. Although evidence supported a key role for inflammation-neuroinflammation^10^, efficacy of anti-inflammatory treatment so far is poor.

Animal studies have contributed to build up a better understanding of this heart-brain interaction. We^11^ and others^12,13^ reported depressive-like behavior in rodents after coronary artery ligation-induced heart failure, which was sensitive to cardiovascular^14^ as well as different types of brain-targeted treatment^15–19^. In addition, decline of cognitive performance was observed in this model^20^. However, the coronary artery ligation model requires major thoracic surgery, and we previously showed that the outcome in this model could be attributed to the combined effect of surgery for coronary artery ligation and effect of the ligation itself^20^. Therefore, in the present study, the isoproterenol (ISO)-induced myocardial infarction model was used^21^. Not many behavioral studies have been performed in this model, but reduced exploratory behavior^22^ and cognitive impairment^23^ suggest relevant behavioral consequences. In a recent study, decreased open field exploration and declined sucrose preference were observed shortly after ISO^24^. In these studies, an imbalanced pro- and anti-oxidant system^22^ or mitochondrial function^23^, rather than neuroinflammation, were observed as underlying mechanism. Moreover, all three behavioral studies were only performed in male rats.

Exercise training is well-known for its positive impact on physical and mental well-being^25^, and this beneficial influence could be associated with anti-inflammatory mechanisms^26^, as well as increased brain derived neurotrophic factor (BDNF) expression^27,28^. The anti-inflammatory properties of exercise could balance the activated inflammation-neuroinflammation and thereby improve cognition and mood^29^. Similarly, exercise may improve cognitive function and mood by counteracting a declined BDNF expression^30^. In the ISO model, beneficial effects of physical exercise were observed^31,32^, but studies mainly focused on cardiac aspects. Nevertheless, exercise training could prevent upregulation of 18 cytokines after ISO^32^, supporting anti-inflammatory potential of exercise training in this model. Exercise training from 12 weeks before ISO injections to 7 days after ISO reduced responses of proinflammatory TNFα and IL6, while IL10 response was increased^33^. However, effects of exercise training on mood and cognition are fairly unexplored in the ISO model.

Aim of the present study was to evaluate the effects of exercise training on heart, brain and behavior in the ISO model in middle-aged female rats.

## Methods

### Animals and experimental design

Forty-seven middle-aged (12 months old) female Wistar rats were obtained from the breeding colony of the University of Physical Education, Hungary. Animals were housed in groups of 2 or 3 in cages of 30 * 42 * 20 cm with sawdust as bedding. Rats were kept in the conventional animal facility of University of Physical Education, Hungary in a room with 22 ± 2 °C and humidity of 50 ± 10%. Light was provided from 7 am to 7 pm CEST. Standard rodent chow (LT/R, Innovo Ltd., Gödöllő, Hungary) and tap water were provided ad libitum. All methods were performed in accordance with the ARRIVE guidelines. All experiments were performed in accordance with relevant guidelines and regulations/legislations. The experiments were conducted under and approved by the general license for animal experiments of the laboratory of Physical Education, University of Budapest, Hungary, under license number TE-KEB/No3/2020.

Rats were randomized to 4 experimental groups; rats were treated with isoproterenol (n = 27) to induce heart lesions, or received saline injections (n = 20), and after one week of recovery, half of the survivors were subjected to five weeks of treadmill running while the other halve received control handling (sedentary) (n = 10 per group). Exploratory behavior and cognitive performance were assessed at the last 7 days of the training period. After completion of all tests, animals were anesthetized and echocardiographic measurements of cardiac function were obtained. Subsequently, rats were sacrificed, and heart and brain tissues were collected for further analyses of cardiac damage, neuroinflammation and neuronal function.

### Cardiac damage

Cardiac damage was chemically induced by isoproterenol hydrochloride (C_11_H_7_NO_3_·HCl: ISO). ISO is a non-selective β-adrenoceptor agonist that mimics the histological, physical and endocrinological events of human myocardial infarction presumably by myocardial hyperactivity induced ischemia and energy depletion^34^. Rats were injected subcutaneously with ISO (Tokyo Chemical Industry Co., Ltd., Tokyo, Japan) in a dose of 70 mg/kg dissolved in 1 ml/kg saline. Control animals received 1 ml/kg saline. Both groups received 2 injections with 24 h in between. The ISO protocol was based on previous studies^23,35^. Two injections of ISO with 24 h in between increased cytokine production leading to cardiac fibrosis^36^, left ventricular hypertrophy and dilatation, and ultimately heart failure^37^.

### Tread-mill running

Both control and ISO treated rats were randomly assigned to treadmill running or sedentary controls. Running was performed on a six-lane rat treadmill (Tartonik Elektronika, Italy) with individual lanes of 12 * 54 * 13 cm. The rats were stimulated to stay on the treadmill by cardboard pushers. No electric shocks were used to motivate the animals. The training program lasted for 5 weeks, 5 times per week on each weekday. On the first week of the training program rats were habituated to running: on the first day, rats started with 10 min of running with a maximal speed of 10 m/min which was gradually increased to 30 min and maximal speed of 18 m/min (moderate intensity; approximately 65% of VO_2_max) by the fifth day. For the following four weeks each running session lasted 30 min^35^.

### Behavior

Behavioral tests were carried out during the last 7 days of the intervention. Interest in environment and anxiety/depressive like behavior was assessed with open field exploration (OF). Regarding cognition, short-term memory was tested in the novel object recognition (NOR) and the novel location recognition test (NLR). All tests were carried out in a quiet and clean test room (between 10 and 12 a.m.) with the same temperature and humidity parameters as the housing room.

All tests were recorded with a digital video camera (Canon Legria HFR106, Canon Inc., Tokyo, Japan) and stored on a memory card for later off-line analyses using Eline software (University of Groningen, the Netherlands).

#### Open field

Open field (OF) exploration test was performed to assess exploratory and anxiety related behavior^11^. A round shaped arena (diameter of 80 cm) was divided into an inner circle (diameter of 32 cm; center area), and an outer annulus (wall area) by black circular lines, and surrounded by a 45 cm tall wall. Animals were placed in the arena and allowed to explore for 5 min. After each animal the arena was cleaned with 70% ethanol to remove smell cues. Locomotor activity was estimated from the number of line crossings. Time spent in the wall area was obtained using Eline software (University Groningen, the Netherlands). More locomotor activity was regarded as more exploration and therefore more interest in the environment. More time in the wall area was regarded as choice for the safe area and therefore sign of anxious-depressive like behavior.

#### Novel object and novel location recognition

The novel object recognition test (NOR) was performed to assess short term visual memory, which depends primarily on prefrontal cortex function^38^, while the novel location recognition test (NLR) determined short term spatial memory, associated with hippocampal activity^38^. The two memory tests were combined in one protocol^38^ and took place in a black box of 45 * 55 * 50 cm. The combined test consisted of 4 phases, each of 3 min, with 1 min in between: (1) in the habituation phase the animal was placed in the test box and allowed 3 min to get accustomed to the settings, (2) in the exploration phase the rat was presented two identical objects, (3) in the novel location phase the two identical objects were presented again but one of them on a different location than in the previous phase, (4) in the novel object phase one of the two identical objects were replaced by a different object and put to the same location as in the preceding phase. Between the phases, the objects were removed and cleaned with 70% ethanol to remove smell cues. After each animal the test box and objects were also cleaned with 70% ethanol. All phases were recorded by a digital video camera (Canon Legria HFR106, Canon Inc., Tokyo, Japan). Time spent with exploring the objects were measured using Eline software (University of Groningen, the Netherlands). Preference for the novel location or the novel object was calculated by dividing the time spent exploring the novel location or novel object by the time spent exploring both objects, while 50% indicated chance level = no recognition. Animals who did not explore the objects or only one of them were excluded at the final statistical analysis.

### Cardiac function

Cardiac function was evaluated with transthoracic echocardiography in a subgroup of rats, randomly chosen from each experimental group, because of limited access to the ECHO machine. Rats were anesthetized with pentobarbital (60 mg/kg, ip), and placed in supine position on heating pads (37 °C core temperature). Standard two-dimensional and M-mode long- and short-axis images at the midpapillary level were acquired using a 13 MHz linear transducer (12L-RS; GE Healthcare, Horten, Norway) connected to a commercially available system (Vivid i; GE Healthcare). Images were analyzed using dedicated software (EchoPac v113; GE Healthcare). Heart rate was obtained. From the images acquired, stroke index (stoke volume indexed to body weight), cardiac index (cardiac output indexed to body weight = stroke index times heart rate) and left ventricular ejection fraction were calculated.

### Tissue collection and processing

At the end of the experiment rats were terminally anaesthetized with 6% sodium pentobarbital solution injected intraperitoneally (2 ml/kg) and perfused transcardially with heparinized (1 ml/l) 0.9% saline. Heart and brain tissues were dissected. Brain and heart tissue was immersion fixated in 4% buffered formaldehyde freshly depolymerized from paraformaldehyde. After 4 days, tissue was washed in 0.01 M phosphate buffered saline (PBS), dehydrated using a 30% sucrose solution, and subsequently quickly frozen in liquid nitrogen and stored at -80 °C until further processing. Microscopical sections were cut. Heart sections were placed on glass immediately after cutting, and processed for histochemical staining of collagen. For brain tissue, free floating sections were stored in 0.01 M (PBS) containing 0.1% sodium azide at 4 °C till further processing for immunohistochemistry. In a subgroup of rats (n = 5–6 per group), randomly chosen from each experimental group, immunohistochemistry staining was performed to visualize microglia, immature neurons (double cortin positive cells) and brain derived neurotrophic factor expression, as has been described previously^38^.

### Immunohistochemistry

#### Cardiac collagen

Since ISO was anticipated to cause focal myocardial infarcts, percentage collagen was used to measure cardiac damage. For that, 25 µm thick transverse slices at mid-ventricular level of the heart were stained with Sirius red (Sigma, Aldrich) and fast green as counterstaining^20^. Colour pictures were taken. Image analysis (Image Pro plus, USA) was used to measure the collagen positive (red) area and was expressed as percentage of total left ventricular tissue area.

#### Microglia

Microglia activity was used as measure for neuroinflammation. To visualize microglia, immunohistochemical staining of ionized calcium binding adaptor molecule 1 (IBA-1) was performed, as described in detail previously^39^. Briefly, after pretreated with 0.3% H2O2 for 20 min., sections were incubated for 3 days with 1:2,500 rabbit-anti IBA-1 (Wako, Neuss, Germany) in 2% bovine serum albumin, 0.1% triton X-100 at 4 °C, followed by a 1 h incubation with 1:500 goat-anti rabbit secondary antibody (Jackson, Wet Grove, USA) at room temperature. The sections were then incubated for 2 h with avidin–biotin peroxidase complex (Vectastain ABC kit, Vector, Burlingame, USA) at room temperature. Labeling was visualized by using a 0.075 mg/mL diaminobenzidine (DAB) solution activated with 0.1% H2O2. All dilutions were made in 0.01 mol/L PBS. All sections were thoroughly rinsed 4 times with 0.01 mol/L PBS between staining steps. Sections were mounted onto glass slides in a 1% gelatin solution and dehydrated through gradients of ethanol and xylol solutions. Photographs were taken from the prefrontal cortex (Pfc), the paraventricular nucleus of the hypothalamus (PVN), and the dorsal hippocampus (hippocampus; CA1, CA3, Dentate Gyrus and Hilus areas) at 200 × magnification. Microglia morphology was analyzed (Image Pro Plus, USA) according to our previous publication^39^, regarding coverage, density, cell size, cell body area and processes area. Microglia activity was calculated as cell body area/total cell size^39^.

#### Neurogenesis

Double Cortin (DCX) staining was used to obtain a measure for hippocampal neurogenesis, as described in detail elsewhere^38^. Images of DCX stained sections of the dentate gyrus of the hippocampus were taken at 50 × magnification. The number of labeled neuronal cell bodies was counted manually by two independent researchers blinded for the experimental groups, and corrected for the length of the DG (Image Pro Plus, USA).

#### Brain-derived neurotrophic factor (BDNF)

For brain function, brain slices were stained with Brain Derived Neurotrophic Factor (BDNF) antibody (Alomone Labs, Israel). In the different areas of the dorsal hippocampus, CA1, CA3, Dendate Gyrus and Hilus, BDNF expression was obtained as corrected optical density (Image-J, USA) compared to an underlying reference area, as described previously^38^.

### Data analysis and presentation

The study has been reported in accordance with ARRIVE guidelines. All reports were performed in accordance with relevant guidelines and regulations/legislations. Data are presented as mean and standard error of mean (SEM), unless indicated otherwise. Results outside twice the standard deviation of its group were considered outliers and were excluded before analyses (maximally 1 per experimental group). Results were compared using two-way analysis of variance (ANOVA) with least square difference (LSD) post-hoc tests, with saline/ISO (indicated as treatment = T) and sedentary/runner (indicated as intervention = I) as factors. Statistically significant outcomes of the two-way ANOVA are presented in the text, while results of post-hoc testing are presented within the figures, indicating significant differences between specific groups. Sample size (n) is mentioned in the figure legends. Association between selected parameters were measured with Pearson linear correlation. For the Novel Object /Novel Location Recognition tests, outcomes were also tested against change level (= 50%), using a single sample t-test. A *p*-value of < 0.05 was considered statistically significant and presented as *. Potentially relevant tendencies (*p* < 0.1) were mentioned as well.

## Results

### General

ISO-induced mortality was 26%, and occurred within 3 days after ISO treatment; that is before separating the groups according to sedentary/exercise intervention. Body weight at the end of the protocol was slightly higher in rats that had performed exercise, independent of saline or ISO treatment (saline sedentary: 253 ± 7 g; saline exercise: 266 ± 9 g; ISO sedentary: 243 ± 7 g and ISO exercise: 264 ± 7 g; n = 10 per group; ANOVA: *p* = 0.141; effect of treatment F_1,39_ = 0.560, *p* = 0.459; effect of intervention F_1,39_ = 4.939, *p* = 0.033; interaction F_3,39_ = 0.320, *p* = 0.575).

### Effects on the heart

**Figure 1:**
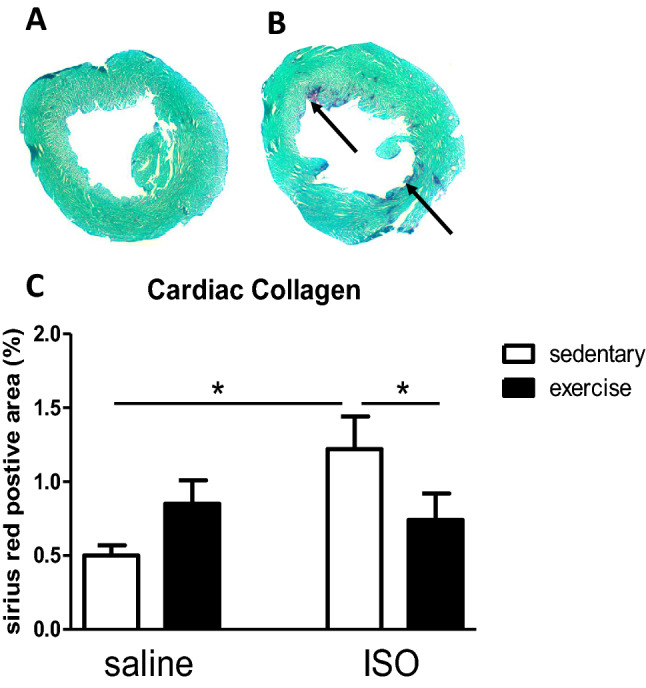
(**A**) and (**B**): typical pictures of sections of the left ventricle, stained for collagen with Sirius red (purple) and fast green as counterstaining. (**A**) section of a saline sedentary rat; (**B**): section of an ISO treated sedentary rats. Arrows point to areas with increased collagen. (**C**): Measured percentage of collagen in the left ventricle as Sirius Red positive area, at mid-ventricular level, in saline or isoproterenol (ISO) treated rats, under sedentary conditions or after five weeks of exercise training (n = 10, 10, 10, 9 for saline sedentary, saline exercise, ISO sedentary and ISO exercise rats, respectively). *: significant difference between groups (*p* < 0.05).

Heart weight to body weight ratio did not differ between groups (saline sedentary: 0.33 ± 0.03%; saline exercise: 0.38 ± 0.01%; ISO sedentary: 0.38 ± 0.02% and ISO exercise: 0.35 ± 0.02%; ANOVA, *p* = 0.237; effect of treatment F_1,39_ = 0.351, *p* = 0.557; effect of intervention F_1,39_ = 0.355, *p* = 0.555; interaction F_3,39_ = 3.724, *p* = 0.062). Figure 1 presents effects on cardiac damage, measured as percentage of collagen at mid-ventricular level (effect of treatment F_1,38_ = 3.310, *p* = 0.077; effect of intervention F_1,38_ = 0.158, *p* = 0.693; interaction F_3,38_ = 6.369, *p* = 0.016). ISO significantly increased cardiac collagen, which was significantly reversed by exercise.

**Figure 2:**
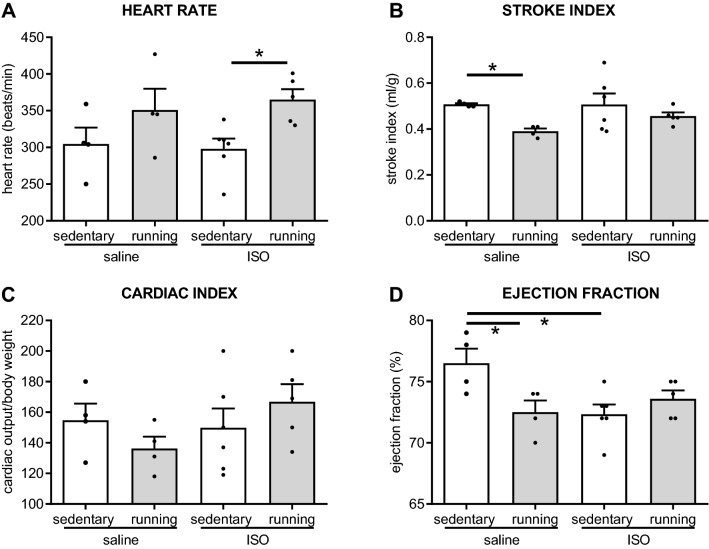
Echocardiographic measures of cardiac function in saline or isoproterenol (ISO) treated rats, under sedentary conditions or after five weeks of exercise training, (n = 4, 4, 6, 5 for saline sedentary, saline exercise, ISO sedentary and ISO exercise rats, respectively). *: significant difference between groups (*p* < 0.05); + : tendency (ANOVA *p* = 0.089; subsequent post-hoc test < 0.05).

Effects on cardiac function were measured by echocardiography. Figure 2 presents the main results. Two-way ANOVA revealed a significant higher heart rate in exercise versus sedentary rats, which appeared most prominent in the ISO treated rats (ANOVA *p* = 0.011; effect of treatment F_1,18_ = 0.034, *p* = 0.856; effect of intervention F_1,18_ = 8.482, *p* = 0.011; interaction F_3,18_ = 0.287, *p* = 0.600). Although analysis of stroke index did not reach statistical significance in the two–way ANOVA (*p* = 0.089), the indicated reduced stroke index after exercise training (post-hoc analysis; *p* = 0.035) may be of physiological relevance (effect of treatment F_1,18_ = 0.914, *p* = 0.354; effect of intervention F_1,18_ = 6.465, *p* = 0.023; interaction F_3,18_ = 0.940, *p* = 0.348). The product of heart rate and stroke index, cardiac index, was preserved in all groups. Left ventricular ejection fraction, as measure for left ventricular function, was significantly declined by exercise training as well as by ISO, but may be partly reversed by combined ISO plus exercise; the latter being not significantly lower than sedentary saline controls anymore (effect of treatment F_1,18_ = 2.745, *p* = 0.118; effect of intervention F_1,18_ = 0.1.931, *p* = 0.185; interaction F_3,18_ = 8.673, *p* = 0.010). Underlying measures of cardiac dimensions are summarized in Supplementary Table 1 None of the cardiac function parameters correlated with collagen percentage.

### Effects on the brain

**Figure 3:**
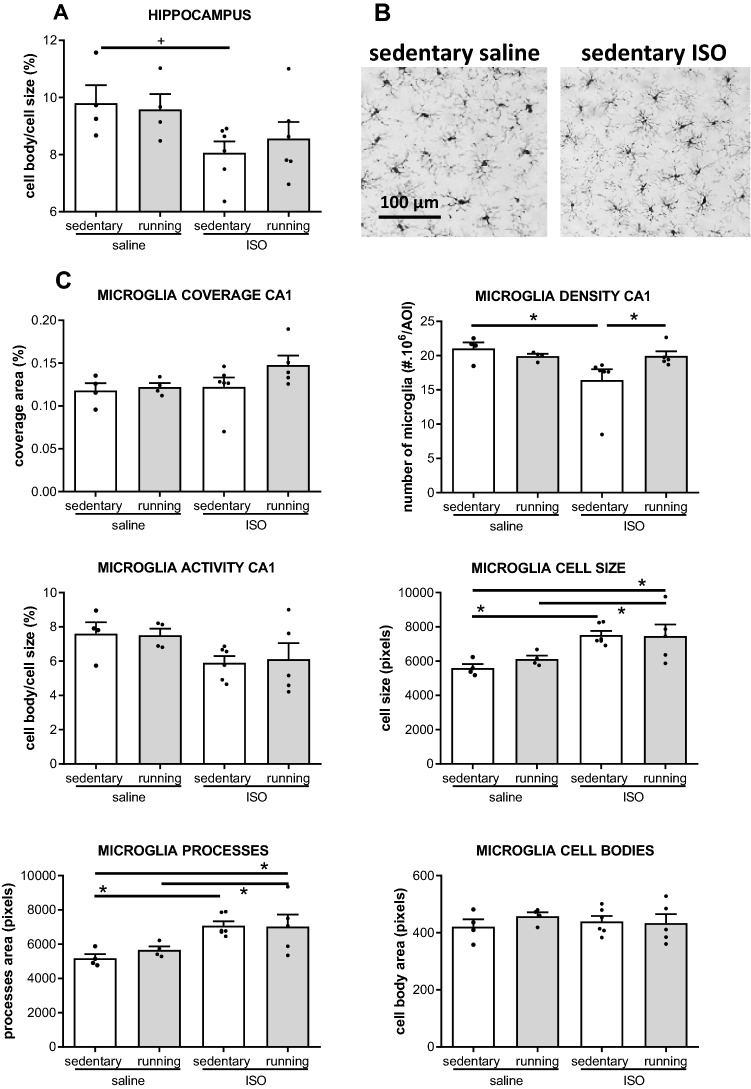
Microglia activity in the hippocampus, in saline or isoproterenol (ISO) treated rats, under sedentary conditions or after five weeks of exercise training, (n = 4, 4, 6, 6 for saline sedentary, saline exercise, ISO sedentary and ISO exercise rats, respectively). (**A**): Overall microglia activity in the hippocampus; (**B**): typical pictures of microglia in the CA1 in saline sedentary and ISO sedentary rats (tile size: 0.1mm^2^); (**C**): Morphological parameters underlying the calculated microglia activity in the CA1 area, as the area best reflecting overall effects on the hippocampus. + : tendency (ANOVA *p* < 0.10; subsequent post-hoc test < 0.05).

Neuroinflammation was obtained from morphological changes of microglia, indicated as microglia activity; cell body to cell size ratio. Prefrontal cortex microglia activity was not affected by ISO, nor by exercise training (saline sedentary 8.0 ± 0.6; saline exercise 8.6 ± 0.6; ISO sedentary 7.5 ± 0.7; ISO exercise 8.3 ± 1.2). Similarly, no significant effects were observed in the paraventricular nucleus of the hypothalamus microglia activity (saline sedentary 9.5 ± 1.1; saline exercise 7.5 ± 0.2; ISO sedentary 7.1 ± 0.8; ISO exercise 8.0 ± 1.7). Moreover, none of the parameters of microglia morphology in these areas appeared affected by either ISO, or exercise, or showed interaction. Nevertheless, microglia activity in the PVN seemed reduced by about 50% after ISO. Similarly, ISO substantially reduced microglia activity in the hippocampus; an effect that was not affected by exercise (Fig. 3) (effect of treatment F_1,19_ = 6.290, *p* = 0.023; effect of intervention F_1,19_ = 0.063, *p* = 0.805; interaction F_3,19_ = 0.435, *p* = 0.519). Overall hippocampal parameters for microglia morphology were not different between groups, as ISO increased coverage by only 5%, but microglia cell size increased by 44%, which was mainly attributable to increased processes (47%), as cell bodies increased only 15%. Exercise training did not affect these observations.

Since the hippocampus plays a role in mood as well as cognition, effects on the different hippocampal areas were analyzed as well to evaluate local differences (see Fig. 3 and Table 1). What was indicated for overall values of the hippocampus, appeared merely reflected in the CA1 area (Fig. 3). ISO decreased microglia density (ANOVA *p* = 0.043; effect of treatment F_1,18_ = 3.823, *p* = 0.069; effect of intervention F_1,18_ = 0.000, *p* = 1.000; interaction F_3,18_ = 3.839, *p* = 0.069), and increased the size of the microglia (ANOVA *p* = 0.010; effect of treatment F_1,18_ = 15.290, *p* = 0.001; effect of intervention F_1,18_ = 0.311, *p* = 0.585; interaction F_3,18_ = 0.484, *p* = 0.497), hence preserving coverage. Increased cell size could be attributed to increased processes (ANOVA *p* = 0.014; effect of treatment F_1,18_ = 14.259, *p* = 0.002 effect of intervention F_1,18_ = 259, *p* = 0.623; interaction F_3,18_ = 0.387, *p* = 0.543), as cell body size remained unaltered; microglia hyper-ramification. In the other hippocampal areas, coverage increased in exercise after ISO, compared to exercise after saline treatment, without significantly altering morphology of the individual microglia.

**Table 1:** Morphological parameters of microglia in different areas of the hippocampus CA3, Dentate Gyrus (DG) and Hilus), in saline or isoproterenol (ISO) treated rats, under sedentary conditions or after five weeks of exercise training (n = 4, 4, 6, 6 for saline sedentary, saline exercise, ISO sedentary and ISO exercise rats, respectively).

Experimental group/brain area	Saline sedentary	Saline exercise	ISO sedentary	ISO exercise
**CA3**				
Density (#/area)	27.8 ± 1.1	24.3 ± 1.6	25.2 ± 0.7	32.4 ± 7.9
Coverage (%)	13.3 ± 0.7	11.4 ± 0.6	13.9 ± 0.4	**14.8 ± 0.9#**
Cell size (pixel)	4798 ± 176	4754 ± 324	5534 ± 216	5264 ± 615
Cell body size (pixel)	435 ± 6	430 ± 12	443 ± 30	426 ± 33
Processes size (pixel)	4363 ± 171	4324 ± 312	5091 ± 216	4838 ± 588
Activity (%)	9.1 ± 0.3	9.1 ± 0.4	8.1 ± 0.6	8.5 ± 0.7
**DG**				
Density (#/area)	27.5 ± 2.5	25.1 ± 0.4	24.2 ± 1.1	25.7 ± 1.2
Coverage (%)	13.1 ± 0.5	14.6 ± 0.7	22.2 ± 0.9	**16.5 ± 1.1 + + $**
Cell size (pixel)	4875 ± 444	5493 ± 353	5936 ± 593	6491 ± 495
Cell body size (pixel)	459 ± 12	422 ± 34	432 ± 16	434 ± 38
Processes size (pixel)	4416 ± 439	5071 ± 385	5504 ± 585	6058 ± 482
Activity (%)	9.7 ± 1.0	7.9 ± 1.0	7.6 ± 0.6 +	6.8 ± 0.6$
**Hilus**				
Density (#/area)	39.6 ± 1.3	37.2 ± 1.4	33.8 ± 1.2	44.1 ± 5.9
Coverage (%)	11.8 ± 0.4	11.5 ± 0.8	12.8 ± 1.0	**14.7 ± 0.5# + $**
Cell size (pixel)	3006 ± 196	3087 ± 196	3805 ± 319	3525 ± 279
Cell body size (pixel)	382 ± 10	417 ± 22	395 ± 18	413 ± 14
Processes size (pixel)	2624 ± 191	2670 ± 212	3410 ± 312	3112 ± 296
Activity (%)	12.9 ± 0.8	13.8 ± 1.6	10.7 ± 0.9	12.1 ± 0.9

*Significant effect of ISO compared to respective saline groups (*p* < 0.05); #: significant effect of exercise training compared to respective sedentary groups (*p* < 0.05); $: significant effect of combined ISO and exercise, compared to saline sedentary (*p* < 0.05); + : potentially relevant effect 0.05 < * p* < 0.1. Significant values are in bold.

**Figure 4:**
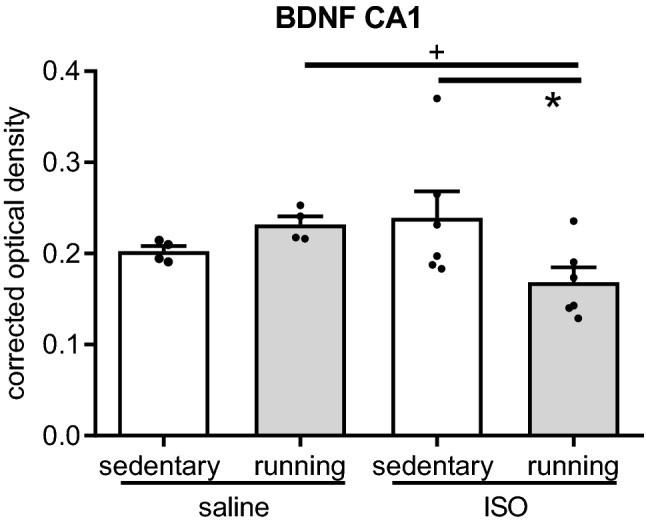
Expression of BDNF in the CA1 region of the hippocampus in saline or isoproterenol (ISO) treated rats, under sedentary conditions or after five weeks of exercise training, (n = 4, 4, 6, 6 for saline sedentary, saline exercise, ISO sedentary and ISO exercise rats, respectively). OD = optical density; *: significant difference between groups (*p* < 0.05); ^+^: *p* = 0.052.

No significant effects were observed on neurogenesis, nor on overall hippocampal or DG BDNF expression (Supplementary Table 2). However, although 2-way ANOVA appeared to show a trend in the CA1 area (*p* < 0.1), but not in other areas, BDNF was significantly decreased by exercise in ISO rats (Fig. 4) (effect of treatment F_1,19_ = 0.386, *p* = 0.543; effect of intervention F_1,19_ = 0.922, *p* = 0.351; interaction F_3,19_ = 5.506, *p* = 0.032).

### Effects on behavior

**Figure 5:**
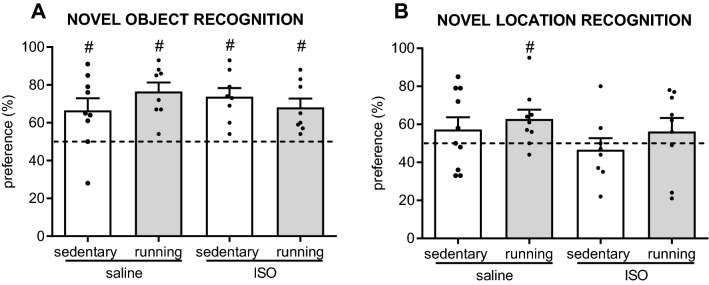
Short-term memory tested as recognizing a novel object (NOR) or a novel location (NLR) in saline or isoproterenol (ISO) treated rats, under sedentary conditions or after five weeks of exercise training, (NOR: n = 9, 9, 8, 8 for saline sedentary, saline exercise, ISO sedentary and ISO exercise rats, respectively; NLR: n = 10, 9, 8, 9 for saline sedentary, saline exercise, ISO sedentary and ISO exercise rats, respectively). #: significantly different from random performance (= 50%; dashed line).

Effects on short-term memory were obtained from performance in the NOR and NLR tests (Fig. 5). For the NOR test, data of six rats were excluded because they did not meet the degree of exploration criteria (four with no exploration and 2 with exploration of only one object), resulting in 8–9 rats per experimental group. For the NLR test, four rats were excluded for only exploring one object, resulting in 8–10 rats per group. The rats that were excluded did not happen to be the most anxious rats in the open field. No significant differences were observed between the experimental groups in performances in these cognitive tests. Twelve months old female rats were well capable of recognizing the novel object in the NOR test, irrespective of saline/ISO treatment or sedentary/exercise training. In the NLR test, control saline sedentary rats seemed unable to recognize the relocated object. Exercise training may improve that, as saline-treated exercise rats performed above random level. However, exercise in ISO-treated rats did not improve NLR performance.

**Figure 6:**
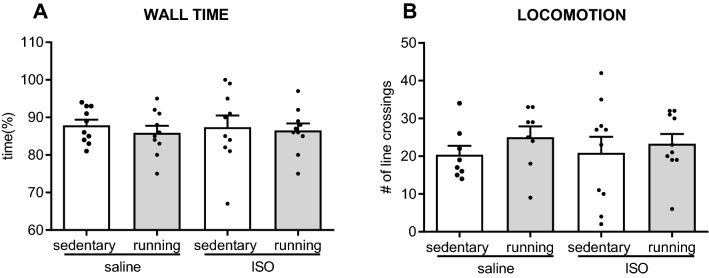
Behavior in the open field test (n = 10 per group), as measured time spent in the wall area and locomotor activity, in saline or isoproterenol (ISO) treated rats, under sedentary conditions or after five weeks of exercise training. *: significant difference between groups (*p* < 0.05).

Effects on mood were indicated by spatial exploration in the OF (Fig. 6). Locomotor activity was not affected by ISO, nor by exercise. Spatial exploration in the OF, measured as wall time, revealed no significant effects of ISO, nor of exercise.

## Discussion

### General

Aim of the present study was to evaluate the effects of exercise training on heart, brain and behavior in the ISO model in middle-aged female rats. As expected, ISO induced cardiac damage and declined cardiac function. However, these effects could not be associated with neuroinflammation, reduced hippocampal neurogenesis or impaired neuronal function. Accordingly, no signs of cognitive impairment or depressive-like behavior were observed after ISO. Exercise training reversed cardiac damage and partly restored ISO-induced cardiac dysfunction, but left neuroinflammation and behavior merely unaltered. Hence, our data indicated that the cardiac damage after ISO in middle-aged female rats, and the subsequent beneficial effects of five weeks exercise training on the heart, were not reflected in changes in the brain nor in altered behavior.

### Effects of ISO

The ISO model has a long history as a method to induce focal cardiac damage and consequently cardiac dysfunction^40^. Similar to our set-up, two injections of ISO given 24 h apart mimicked acute sympathetic stress, activating the innate immune system, resulting in focal cardiac necrosis shown by collagen deposition^32,36^, that over a period of weeks developed into heart failure^37^. In contrast to Grant et al.^41^, other researchers indicated sex difference in the ISO model, as male rats had a lower survival rate, developed more massive necrosis and displayed slower repair compared to female rats^34,42^. Accordingly, when compared to our previous study in male rats of the same age^35^, in the female rats in the present study cardiac fibrosis and mortality were relatively mild. Nevertheless, cardiac fibrosis was eminent and caused long-term cardiac dysfunction, suggesting activation of inflammation as repair mechanism. However, these effects could not be associated with neuroinflammation (microglia activation) or associated behavioral changes, such as cognitive decline or depressive-like behavior 5 weeks later. In fact, in contrast to our previous study in male rats^35^, in female rats microglia activity in the hippocampus indicated reduced, rather than elevated neuroinflammation after ISO. Usually, injury evokes “classical” pro-inflammatory activation of microglia, with morphological changes that include shortened dendrites with increased cell body size^43^. However, alternatively activated microglia show opposite morphological changes, and are associated with non-pathological stimuli^44,45^. In our study, we observed a significant increase in total microglial cell size, that could be attributed to increased dendrite area of microglia cells, as cell body area remained unchanged. This finding indicated that the microglia converted into the alternatively activated phenotype, associated with microglia priming; microglia sensitization to become hyperactive once (re)activated^43^.

Since women seem more susceptible to develop depression after myocardial infarction, we anticipated that our female rats also may develop signs of depressive-like behavior after ISO. However, we did not observe altered OF behavior; neither less locomotion, nor more time in the safe wall area, hence no signs of depressive-like behavior in our female ISO rats. Although depressive-like behavior has been described in literature for male rats, and shortly after ISO-induced damage^22,24^, in our previous study 5 weeks after ISO in male rats^35^, if anything, ISO seemed to reduce depressive-like behavior.

To our knowledge, no other female rat studies on behavior are available in that regard. Nevertheless, the older studies of Wexler and coworkers^46,47^ clearly indicated sex differences in the response of peripheral parameters, that could be linked to behavioral changes, such as depression and cognition^48^.

### Effects of exercise

Exercise training is well recognized for it is beneficial effect on physical as well as mental health^25^. Indeed, exercise training reversed the ISO-induced scar formation in the heart, thereby partly restoring left ventricular function in our female rats. However, in our previous study in male rats, no cardiac effects of exercise were observed^35^. Actually, in the only study we are aware of that started endurance training after-, instead of before ISO, exacerbated cardiac damage was reported after four weeks of training^49^. Differences with the present study may be attributed to the age; 8–10 weeks versus 12 months, and sex of the rats, male- versus female rats, as well as the time point of start of the exercise training; two days after ISO, compared to the one-week delay in our study. Especially the latter aspect could provide an explanation. Our study was set-up to intervene when cardiac damage was eminent, at one week after ISO, whereas the study of Jazi et al.^49^ started exercise during the inflammatory healing phase, with no cardiac fibrosis present yet^32^. In the present study exercise intervention could then have been started when the acute healing process was merely complete, hence interfering with the more chronic inflammatory state; timing is crucial.

Exercise training is indicated to exert it beneficial effects, amongst others, by its anti-inflammatory action^26^. Since we hypothesized that depressive-like behavior^9^ as well as cognitive impairment^20^ in association with myocardial infarction, are at least in part attributable to a derailed peripheral inflammation-neuroinflammatory response, we anticipated increased neuroinflammation after ISO, which would be reversed by exercise training. Since we did not observe increased neuroinflammation, measured by microglia activity, nor depressive-like behavior or cognitive decline after ISO, accordingly, we were unable to determine potential reversal of these effects by exercise training. Although neuronal function, as indicated by altered BDNF levels, was not declined after ISO, results seemed to indicate a decline after exercise in ISO rats. These observations are in contrast to our expectations; that were a decline after ISO, that could be reversed by exercise. Brain-derived neurotrophic factor (BDNF) has an important role in regulating maintenance, growth and survival of neurons. It is long known that exercise training increases BDNF expression in the hippocampus^50^, playing a beneficial role in learning and memory^51^. However, circulating BDNF also increases after exercise, which could be mainly attributed to the increased brain expression (hippocampus and cortex)^52^. Moreover, exercise training after myocardial infarction in male rats also induced BDNF in skeletal muscle and the non-infarct area of the left ventricle, which may contribute to improvement of muscle function as well as cardiac function^53^. The declined BDNF expression in the hippocampus (CA1 area) in the present study, in contrast to the increase seen in male rats in our previous study^35^, would then be contradictory to the improved cardiac aspects after exercise in ISO rats, and may well related to the female sex of the rats.

### Timing

An important aspect of intervention is timing. As we aimed to intervene with the ISO-induced cardiac disease when cardiac fibrosis was eminent, we started exercise training one week after ISO. According to Alemasi et al.^32^ cardiac inflammation is progressing 3 days after ISO, while cardiac fibrosis seemed not present before seven days after ISO. All referred studies on exercise intervention that had started exercise training before ISO, showed improvement of cardiac parameters, potentially interfering with the inflammatory and/or metabolic responses early after ISO. Our previous study on exercise intervention starting one week after ISO in male rats, showed no effect on cardiac collagen percentage^35^. However, the one study that started exercise training two days after ISO^49^, that is within the inflammation phase^32^, even showed deterioration of cardiac outcome. These results may indicate that optimal timing of exercise intervention in relation to the time course of the inflammatory/metabolic response to ISO seemed crucial. Since in the present study, we showed a positive effect of exercise on the heart, without clear consequences for brain function, this may add to sex differences in the ISO-induced pathology. Striking finding was the indicated microglia hyper-ramification after ISO in female rats. Acute ISO administration produced tachycardia associated with relative ischemia due to imbalance between increased myocardial oxygen demand and reduced coronary blood supply^37^. Another study, evaluating microglia activity four days after an acute imbalance between oxygen supply and demand due to anesthesia, also showed microglia hyper-ramification, that was explained as a prolonged state of priming^54^. Hence, it could well be that one week after ISO, the acute response to ISO is merely over, leaving only limited scope for effects of exercise training. The microglia hyper-ramification six weeks after ISO may then represent long-term alertness, that is still present after exercise intervention. Alternatively, the time course of the inflammatory response on microglia activation, as we have observed after a different inflammatory event (surgery^38^;) could provide an explanation for our results; early increased microglia activity, followed by normalization, and subsequent even significantly declined microglia activity. Therefore, in our opinion, in order to obtain beneficial effects of exercise intervention after ISO, timing of intervention with respect to the time course of cardiac pathology, is crucial.

### Limitations

Although the set-up was chosen carefully, each study has its limitations. Since we used inflammation-neuroinflammation-depression/cognitive impairment as our working hypothesis, in hindsight it would have been wise to have collected timed blood samples, to further elucidate on the underlying mechanism of our findings, based on plasma markers, TNFα and IL6^33^, or lipocalin^55^. The same holds true for measurements of cardiac inflammatory markers at sacrifice. Although the initial event, the cardiac damage, as well as the ultimate behavioral consequences were measured in all rats, the in between factors were only obtained in subgroup of rats, limiting power of the conclusions concerning mechanisms.

The dose of ISO was selected based on effect (mortality) in our previous study^35^. Actually, this dose appeared in between the medium-dose and high-dose of ISO as reviewed by Nichtova et al.^37^, with potentially mixed effects described for these dose ranges.

Finally, although the ISO model is mainly used to induce cardiac damage, one has to keep in mind that ISO-effects may not be limited to the heart, as indicated by changes in adrenal gland weight and corticosterone levels as well^47^.

## Conclusion

Our data indicated that five weeks of exercise training in middle-aged female rats, starting when ISO-induced cardiac damage was eminent, decreased cardiac damage and partly restored cardiac dysfunction. However, since ISO could not be associated with neuroinflammation, nor with depressive-like behavior and cognitive impairment, anticipated reversal by exercise could not be observed. Hence, our data indicated that the cardiac damage after ISO in middle-aged female rats, and the subsequent beneficial effects of five weeks exercise training on the heart, were not reflected in changes in the brain nor in altered behavior.

## Supplementary Information

Supplementary Information.

## Data Availability

The datasets used and/or analysed during the current study available from the corresponding author on reasonable request.

## Abbreviations

BDNF: Brain-derived neurotrophic factor
CA: Cornu ammonis
EF: Ejection fraction
ISO: Isoproterenol
Iba-1: Ionized-binding adaptor protein 1
LSD: Least square difference
NLR: Novel location recognition test
NOR: Novel object recognition test
OF: Open field
SEM: Standard error of the mean
VO_2_max: Maximal oxygen uptake capacity
