# Supplementary material for: The effects of exercise training on heart, brain and behavior, in the isoproterenol-induced cardiac infarct model in middle-aged female rats
Source: Sci Rep. 2022 Jun 16;12:10095. doi: 10.1038/s41598-022-14168-z (PMC9203707; doi:10.1038/s41598-022-14168-z)
Supplement: Supplementary file 1 — Supplementary Information. [file 41598_2022_14168_MOESM1_ESM.docx]

Supplementary table 1. Table 2. Overall hippocampal BDNF expression, and BDNF- and double cortin expression (neurogenesis) in the Dentate Gyrus of the hippocampus in saline or isoproterenol (ISO) treated rats, under sedentary conditions or after five weeks of exercise training. OD=optical density

| Experimental group | Saline sedentary | Saline exercise | ISO sedentary | ISO exercise |
| --- | --- | --- | --- | --- |
| n | 4 | 4 | 5 | 6 |
| Hippocampus BDNF (OD) | 0.18±0.02 | 0.19±0.01 | 0.18±0.02 | 0.15±0.01 |
| DG BDNF (OD) | 0.22±0.03 | 0.21±0.02 | 0.20±0.02 | 0.19±0.01 |
| DG neurogenesis (#cells/length) | 1.71±0.82 | 0.76±0.28 | 1.40±0.30 | 1.75±0.90 |

Supplementary table 2. Left ventricular dimensions obtained from echocardiography, and calculated fractional shortening in saline or isoproterenol (ISO) treated rats, under sedentary conditions or after five weeks of exercise training. *: significantly different from saline sedentary rats (p,0.05).

| Experimental group | Saline sedentary | Saline exercise | ISO sedentary | ISO exercise |
| --- | --- | --- | --- | --- |
| n | 4 | 4 | 6 | 5 |
| Left ventricular end-diastolic diameter | 5.87±0,12 | 5.56±0.17 | 5.84±0.13 | 5.88±0.14 |
| Left ventricular end-diastolic volume | 171.5±8.1 | 151.4±5.2 | 169.9±8.3 | 172.3±9.1 |
| Left ventricular end-systolic diameter | 3.19±0.07 | 3.22±0.06 | 3.39±0.07 | 3.34±0.10 |
| Left ventricular end systolic volume | 40.7±2.0 | 41.7±1.7 | 47.1±2.1 | 45.8±3.3 |
| Left ventricular mass per body weight | 2.33±0.09 | 2.14±0.02 | 2.37±0.12 | 2.15±0.08 |
| Fractional shortening | 45.6±1.2 | 42.1±0.7* | 42.0±0.7* | 43.2±0.5* |
